# Prospective multicentre evaluation and refinement of an analysis tool for magnetic resonance spectroscopy of childhood cerebellar tumours

**DOI:** 10.1007/s00247-018-4182-0

**Published:** 2018-07-30

**Authors:** Karen A. Manias, Lisa M. Harris, Nigel P. Davies, Kal Natarajan, Lesley MacPherson, Katharine Foster, Marie-Anne Brundler, Darren R. Hargrave, Geoffery S. Payne, Martin O. Leach, Paul S. Morgan, Dorothee Auer, Tim Jaspan, Theodoros N. Arvanitis, Richard G. Grundy, Andrew C. Peet

**Affiliations:** 1 Institute of Cancer and Genomic Sciences, University of Birmingham, Birmingham, UK; 2 Birmingham Children’s Hospital, Birmingham, UK; 3 Department of Radiological Science, Brighton and Sussex University Hospitals NHS Trust, Brighton, UK; 4 Medical Physics and Imaging, University Hospital Birmingham, Birmingham, UK; 5 Paediatric Oncology Unit, Great Ormond Street Hospital, London, UK; 6 Royal Marsden Hospital, Sutton, Surrey, UK; 7 CRUK Cancer Imaging Centre, Institute of Cancer Research and Royal Marsden Hospital, London, SW7 3RP UK; 8 Medical Physics, Nottingham University Hospitals, Nottingham, UK; 9 Radiological and Imaging Sciences, University of Nottingham, Nottingham, UK; 10 Radiology Department, University Hospital Nottingham, Nottingham, UK; 11 Institute of Digital Healthcare, WMG, University of Warwick, Warwick, UK; 12 The Childhood Brain Tumour Research Centre, The Medical School, University of Nottingham, Nottingham, UK

**Keywords:** Brain, Cerebellum, Children, Diagnosis, Magnetic resonance spectroscopy, Tumuor

## Abstract

**Background:**

A tool for diagnosing childhood cerebellar tumours using magnetic resonance (MR) spectroscopy peak height measurement has been developed based on retrospective analysis of single-centre data.

**Objective:**

To determine the diagnostic accuracy of the peak height measurement tool in a multicentre prospective study, and optimise it by adding new prospective data to the original dataset.

**Materials and methods:**

Magnetic resonance imaging (MRI) and single-voxel MR spectroscopy were performed on children with cerebellar tumours at three centres. Spectra were processed using standard scanner software and peak heights for N-acetyl aspartate, creatine, total choline and myo-inositol were measured. The original diagnostic tool was used to classify 26 new tumours as pilocytic astrocytoma, medulloblastoma or ependymoma. These spectra were subsequently combined with the original dataset to develop an optimised scheme from 53 tumours in total.

**Results:**

Of the pilocytic astrocytomas, medulloblastomas and ependymomas, 65.4% were correctly assigned using the original tool. An optimized scheme was produced from the combined dataset correctly assigning 90.6%. Rare tumour types showed distinctive MR spectroscopy features.

**Conclusion:**

The original diagnostic tool gave modest accuracy when tested prospectively on multicentre data. Increasing the dataset provided a diagnostic tool based on MR spectroscopy peak height measurement with high levels of accuracy for multicentre data.

## Introduction

^1^H magnetic resonance (MR) spectroscopy measures the concentration of metabolites in a preselected volume of tissue^.^ Although numerous studies in adults and a smaller number in children have shown MR spectroscopy metabolite profiles to be helpful in the noninvasive assessment of brain tumours [1–12], the technique is currently not widely used in a clinical paediatric setting.

MR spectroscopy is difficult to analyse and its application is hindered by the lack of straightforward analysis tools. The majority of published studies have been based on sophisticated data analysis techniques, including diagnostic classifiers [7, 11, 13, 14] and computerised pattern recognition [9–11, 15–17], which are not readily available to radiologists. In a clinical setting, a fast, simple and easily implemented method of data analysis would be of major benefit to make MR spectroscopy interpretation readily accessible to clinicians without extensive knowledge about spectroscopy or dedicated computer software.

MR spectroscopy can facilitate noninvasive diagnosis as different brain tumour types display characteristic chemical profiles [9]. Quantitative interpretation of individual metabolites may determine tumour type and grade. Brain tumours are characterized by high levels of total choline and lactate and low levels of N-acetyl aspartate [11, 13], with high-grade tumours having high total levels of choline, lipids [18] and glycine [19]. Taurine is often present in primitive neuroectodermal tumours [20] and medulloblastomas [10, 11]. Pilocytic astrocytomas generally display lower levels of creatine than other paediatric brain tumours and have high levels of lactate despite their benign nature [10, 21]. Interpreting MR spectroscopy using sophisticated pattern recognition techniques, such as principal component analysis, linear discriminant analysis and artificial neural networks [3, 17], has been found to be highly accurate in brain tumour classification.

It is important to develop and evaluate simple and robust analysis tools to allow implementation of MR spectroscopy in a routine clinical setting. A recent retrospective study found that including visual interpretation of MR spectroscopy in the preoperative diagnosis of paediatric brain tumours significantly improved accuracy of radiologic diagnosis over MRI alone [22]. This is encouraging as it suggests MR spectroscopy may be used clinically without sophisticated decision support software.

We previously developed an analysis tool based on peak height ratio calculation that accurately classified cerebellar tumours using retrospective data [23], with the aim of providing a simple but robust technique for radiologists to use in a clinical setting. The tool uses standard scanner manufacturer’s software to produce an MR spectrum, followed by peak height measurement and a straightforward set of rules to predict histopathology. This tool correctly classified 26 of 27 spectra from childhood cerebellar tumours, with agreement of 92.5% among 4 blinded testers. There were, however, limitations in that the dataset was small, with ependymomas poorly represented, and data were processed on a single dedicated scanner in one centre.

Despite the increase in studies utilizing MR spectroscopy in diagnosing brain tumours, there are few published prospective studies evaluating analysis tools. Prospective evaluation is vital to determine true accuracy and provide information to allow incorporation of techniques into routine clinical practice. It is difficult to ascertain generalisability of single-centre studies. Prospective testing on a multicentre dataset will enable full evaluation, assess robustness to change of data source and reduce bias. Acquiring a larger dataset may allow refinement and expansion of the method.

The aim of this study was twofold: to prospectively determine the diagnostic accuracy of the previously developed peak height measurement tool in a multicentre setting and to optimize the tool by adding newly acquired data to the original dataset.

## Materials and methods

Data were accrued from patients presenting with cerebellar lesions at one of three centres: Birmingham Children’s Hospital, Queens Medical Centre, Nottingham, and the Royal Marsden Hospital. All data were collected on 1.5-T clinical MR systems, with scanners from Siemens (Siemens Avanto 1.5 T; Siemens Healthcare, Erlangen, Germany), General Electric (GE Signa EXCITE 1.5 T; GE Healthcare, Chicago, IL) and Philips (Philips Ingenia 1.5 T; Philips Healthcare, Best, the Netherlands). Ethical approval was obtained from the East Midlands–Derby Research Ethics Committee (REC 04/MRE04/41) and parental consent was obtained. All scans were performed at diagnosis, before surgery, chemotherapy or radiotherapy.

**Fig. 1:**
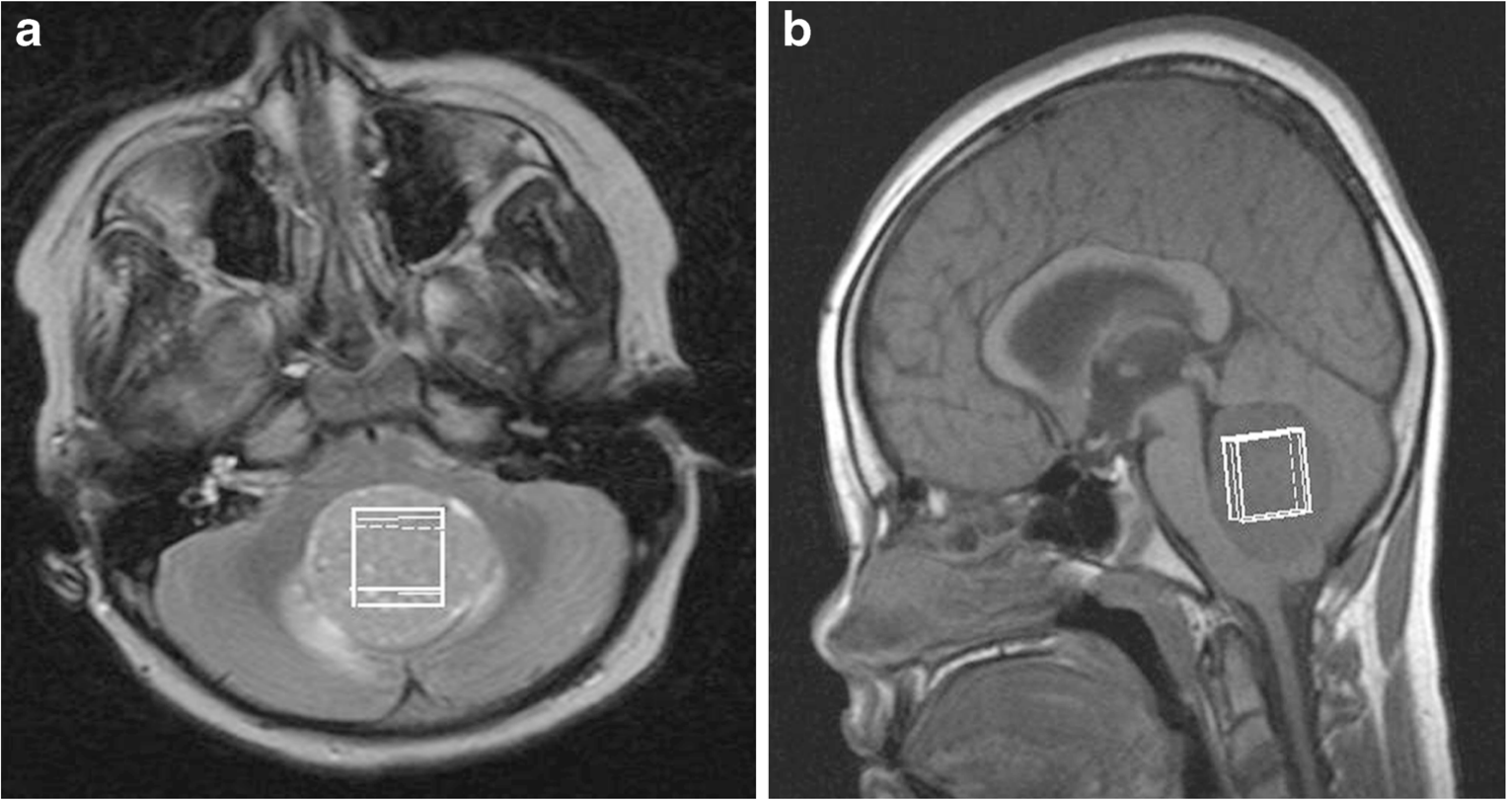
A 7-year-old girl with a midline cerebellar tumour. Axial (**a**) and sagittal (**b**) MR images demonstrate positioning (*white rectangles*) for single-voxel MR spectroscopy acquisition

Single-voxel MR spectroscopy was performed using a standard point resolved spectroscopy (PRESS) sequence with a short echo time (28–35 ms) and a relaxation time of 1,500 ms. A cubic voxel of side length 1.5 or 2 cm was placed entirely within the solid component of the tumour, avoiding cystic regions and areas of haemorrhage or calcification (Fig. 1). One hundred twenty-eight acquisitions were used for the larger voxels and 256 for the smaller ones. MR spectroscopy was processed locally using built-in scanner software, and quality control was applied as in the original study [23]:

1. MR spectroscopy peaks must be sufficiently narrow for the creatine (3.0 ppm [ppm]) and total choline (3.2 ppm) peaks to be visually well separated.
2. The choline peak height must be at least five times greater than the level of noise.

**Fig. 2:**
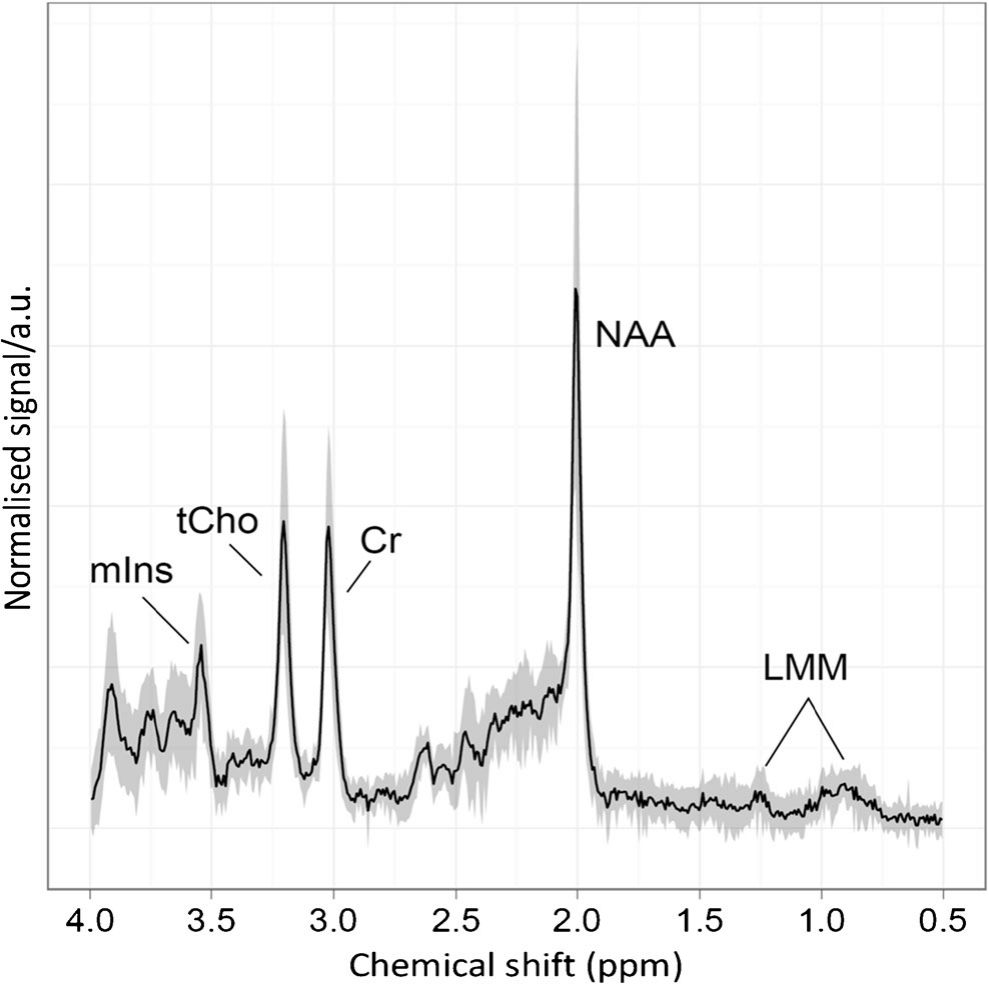
An example ^1^H MR spectroscopy profile of normal brain (white matter) acquired at 1.5 T shows metabolite peaks used in the analysis. *a.u* arbitrary units, *Cr* creatine, *LMM* lipids and macromolecules, *mIns* myo-inositol, *NAA* N-acetyl aspartate, *ppm* parts per million, *tCho* total choline

As in the original classification scheme, peak heights were measured using spectra produced by scanner software. Post-processing was taken as that used routinely for clinical work within each centre, and this was usually within the default scanner settings. Spectra at Birmingham Children’s Hospital were generated by standard Siemens Symphony NUM4 scanner software (Siemens, Erlangen, Germany), which assigns peaks for total choline, creatine, N-acetyl aspartate and myo-inositol. Processed spectra from the Siemens Symphony at Birmingham Children’s Hospital were viewed using imageJ™, a standard Digital Imaging and Communications in Medicine viewer available at http://rsb.info.nih.gov/ij/. For data from other scanners, a screen capture was taken of the spectrum, which was saved as a jpeg to be analysed using the same method. In addition to the major metabolites measured in the previous study (myo-inositol, total choline, creatine and N-acetyl aspartate), peaks associated with resonances due to lipids and macromolecules were measured at both 0.9 and 1.3 ppm. A representative MR spectrum from a normal mature paediatric brain is shown in Fig. 2.

The peak height was taken as the vertical distance between the top of the peak and the baseline. The top of the peak was identified visually as the maximum value within the frequency range giving rise to the peak. Not all spectra had a flat baseline largely due to variation in water suppression, differences in post-processing between scanners and signals from macromolecules. Where the baseline was flat, peak heights were all measured from a set baseline to the apex of each peak. Where the baseline was not flat, the peak height was taken from the base of the individual peak to the apex. Measurements were repeated 3 times by an independent researcher with experience in spectroscopy (L.M.H., with 3 years of experience) to reduce error. Ratios were generated between peak heights for the six peaks measured.

### Classification using original scanner classifier

Cases passing quality control were separated into four groups: pilocytic astrocytoma, ependymoma, medulloblastoma and other, according to histopathological diagnosis. The tumours in the first three groups, i.e. the three tumour classes used in the initial scheme, were assessed using the original classification scheme [23]. Classification rates were determined for prospective data both collected on the Siemens MRI scanner at Birmingham Children’s Hospital and that collected on other scanners.

### Determining the best metabolite ratios for tumour discrimination using the whole dataset

An analysis assessed whether metabolite ratios used in the original scheme remained optimal for distinguishing among the three tumour types for the combined dataset. The methodology closely follows that developed in the previous study [23]. In addition to metabolites used in the original study, peaks associated with lipid and macromolecular signals were measured at 1.3 ppm and 0.9 ppm (lipids and macromolecules 1.3 and lipids and macromolecules 0.9, respectively), and ratios between these and total choline were generated. Analysis of variance determined the significance of differences in the means of all metabolite peak height ratios among the three tumour types. Metabolite ratios with no significant difference (*P*<0.05) among tumour groups were discarded. Receiver operator characteristic (ROC) curves were generated for each of the remaining metabolite ratios for each tumour type. The area under the receiver operator characteristic curve (AUC) was taken to be the best measure of accuracy of the test performed. Scatterplots were created from metabolite ratios showing the best discrimination to check distributions visually. Optimal cutoff values for each of the chosen metabolite ratios were taken as points of maximum sensitivity plus specificity.

Tumours that did not have a diagnosis of pilocytic astrocytoma, medulloblastoma or ependymoma were assessed for their main spectral characteristics and the metabolite ratios compared with those of the other three tumour groups using scatterplots.

### Assignment of peak heights

Analysis of spectral fits in the original dataset showed that in many cases the entire broad peak at 2.0–2.5 ppm was assigned to N-acetyl aspartate. As this broad peak has contributions from lipids, macromolecules, glutamate and glutamine, attribution of the whole peak to N-acetyl aspartate results in gross overestimation of the N-acetyl aspartate peak area. This was particularly evident when there was no narrow N-acetyl aspartate peak at 2.0 ppm. The values of N-acetyl aspartate/creatine obtained in the 30 ms and 135 ms echo time spectra were compared in cases in the original dataset where data had been collected at both echo times to test the validity of attributing a peak height at 2.0 ppm to N-acetyl aspartate. This showed a significant correlation between N-acetyl aspartate/creatine values at the two echo times (*P*<0.01).

## Results

**Fig. 3:**
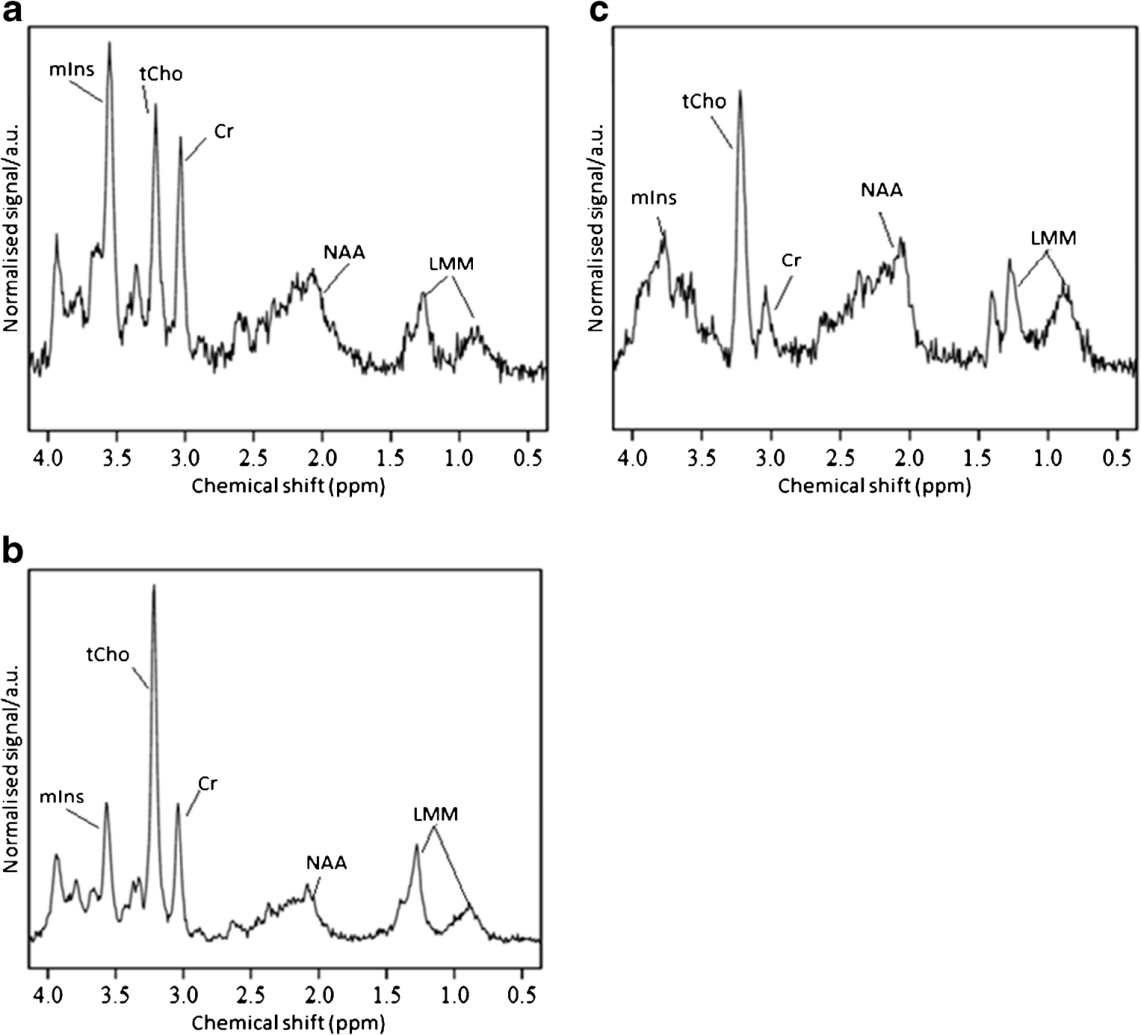
Representative ^1^H MR spectra for the three main tumour types: (**a)** ependymoma, (**b)** medulloblastoma and (**c)** pilocytic astrocytoma. *a.u* arbitrary units, *Cr* creatine, *LMM* lipids and macromolecules, *mIns* myo-inositol, *NAA* N-acetyl aspartate, ppm parts per million, *tCho* total choline

In the new cohort, MRI and short echo time MR spectroscopy were performed on 36 children with cerebellar lesions after Feb. 16, 2005, at three centres. All patients underwent biopsy, leading to a diagnosis of 13 pilocytic astrocytomas, 4 ependymomas, 14 medulloblastomas, 2 atypical teratoid rhabdoid tumors, 1 ganglioglioma, 1 diffuse low-grade astrocytoma and 1 high-grade lesion of undetermined nature. Of these, four pilocytic astrocytomas scanned on the Siemens Symphony at Birmingham Children’s Hospital failed to pass quality control, leaving a group of nine pilocytic astrocytomas for further consideration. In addition, one medulloblastoma case at the Royal Marsden Hospital was excluded due to incomplete data, leaving 13 medulloblastomas in the analysis. A total of 31 patients were included, of which 26 had one of the 3 main tumour types. Representative MR spectra for each of the three main tumour types are shown in Fig. 3.

One spectrum, attributed to a pilocytic astrocytoma, acquired at Queens Medical Centre on a Phillips system, had very poor water suppression and screen capture had removed the lower part of the screen. The spectrum was removed from statistical analysis as peak heights could not be accurately determined, although classification was undertaken to assess whether poor quality data could yield reasonable classification.

### Classification using original scanner classifier

Of the 31 spectra that passed quality control, 26 were of one of the 3 original disease types, leaving 5 spectra from tumours with rare diagnoses not included in the original scheme. Classification rates are summarised in Table 1. The original classifier had a correct classification rate of 65.4% on the new data (*n*=26), correctly classifying 5 of the 9 pilocytic astrocytomass, 2 of the 4 ependymomas and 10 of the 13 medulloblastomas. The original classifier was subsequently applied to the complete dataset of 53 tumours, comprising 26 from the new and 27 from the old cohorts. Overall, this scheme had a classification rate of 81.1% using the complete dataset.

**Table 1:** Classification rates for prospective evaluation of classification scheme for cerebellar tumours, based on peak height measurement from spectra produced by scanner software

Diagnosis	Number of patients	Correct classification (%)	Magnetic resonance spectroscopy classification		
			Pilocytic astrocytoma	Ependymoma	Medulloblastoma
Pilocytic astrocytoma	9	60	5	1	3
Ependymoma	4	50	0	2	2
Medulloblastoma	13	80	3	0	10

### Re-optimising cutoff values

Re-optimizing cutoff values for ratios in the original classifier using a complete dataset of 52 spectra (a pilocytic astrocytoma with poor water suppression was removed from optimization stage) gave values of 2.22 for N-acetyl aspartate/creatine, 0.45 for creatine/total choline and 1.35 for myo-inositol/N-acetyl aspartate, with an overall correct classification rate of 64.2%. The classifier correctly classified 14 of 15 pilocytic astrocytomas, with 4 medulloblastomas and no ependymomas misclassified as pilocytic astrocytomas. The classification accuracy for pilocytic astrocytoma versus the combined group of medulloblastoma and ependymoma was 48/53 (91%). Seven of 8 ependymomas, but only 14 of 29 medulloblastoma spectra, were correctly classified. Of misclassified medulloblastomas, 4 were classified as pilocytic astrocytoma and 11 as ependymoma.

### Determining the best metabolite ratios for tumour discrimination using the whole dataset

**Fig. 4:**
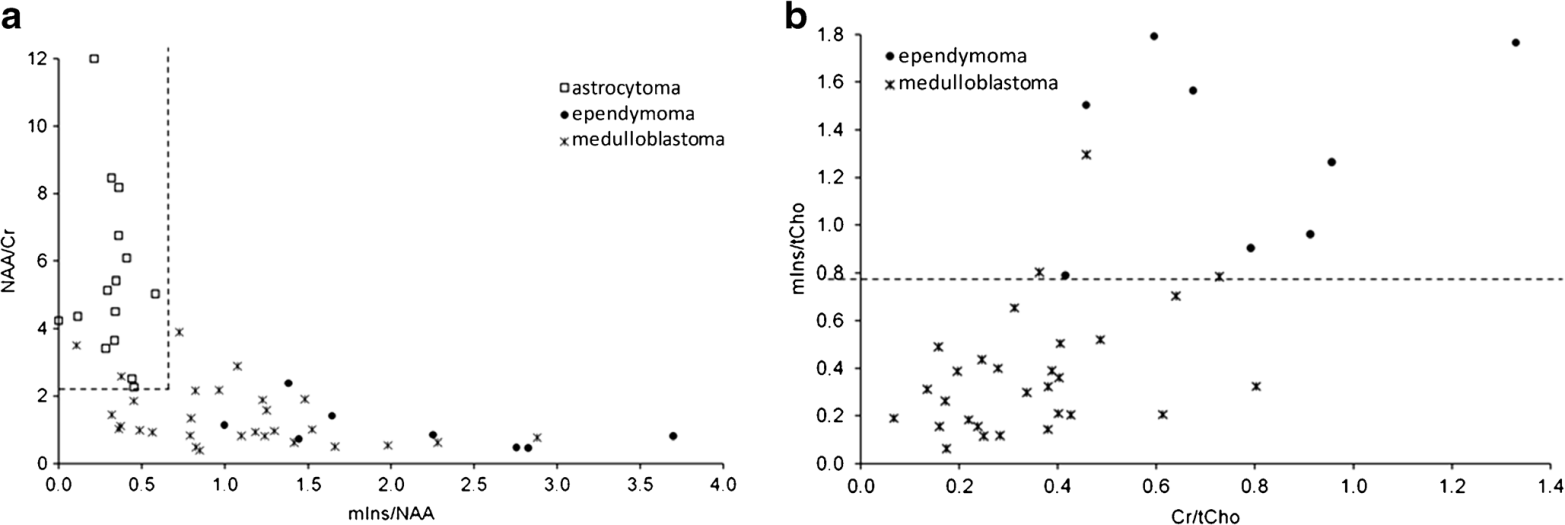
All data from original classification scheme and additional new multicentre data are plotted on classification axes scatterplots. **a** N-acetyl aspartate (NAA)/creatine (Cr) against myo-inositol (mIns)/NAA with the optimum cutoff value for separation of astrocytomas (*white squares*) from ependymomas (*black circles*) and medulloblastomas (*asterisks*) is indicated by horizontal and vertical dashed lines. **b** mIns/total choline (tCho) against Cr/tCho shows the cutoff line required to separate ependymomas (*black circles*) from medulloblastomas (*asterisks*)

A new two-step classifier was developed from the whole dataset as follows. The ratios offering the highest significance separating pilocytic astrocytomas from the other two tumour types were N-acetyl aspartate/total choline, N-acetyl aspartate/creatine and myo-inositol/N-acetyl aspartate, all of which had *P*-values <0.01 (Fig. 4). The ratios offering the most significant difference between ependymoma and medulloblastoma spectra were creatine/total choline and myo-inositol/total choline, both of which had *P*-values <0.01 (Fig. 4). Results of the two-tailed *t*-tests can be seen in Table 2.

**Table 2:** *T*-test results, determining whether mean values of ratios are significantly different between groups, for re-optimising the classification scheme for cerebellar tumours based on spectra from scanner software

	*P*-value	
Ratio	Pilocytic astrocytoma vs. other	Medulloblastoma vs. ependymoma
Cr/tCho	0.785	0.005
NAA/tCho	<0.001	0.019
mIns/tCho	0.272	<0.001
NAA/Cr	<0.001	0.212
mIns/Cr	0.330	0.075
mIns/NAA	<0.001	0.013
LMM 0.9/tCho	0.015	0.184
LMM 1.3/tCho	0.436	0.296
LMM 0.9/LMM 1.3	0.044	0.621

*Cr* creatine, *LMM* lipids and macromolecules, *mIns* myo-insoitol, *NAA* N-acetyl aspartate, *tCho* total choline

Findings were confirmed by calculating the AUC of ROC curves for all ratios with *P*<0.01. AUCs for pilocytic astrocytoma against ependymoma+medulloblastoma (Table 3) revealed 2 ratios with average AUCs greater than 0.9: myo-inositol/N-acetyl aspartate and N-acetyl aspartate/creatine. Both N-acetyl aspartate/creatine and myo-inositol/N-acetyl aspartate were therefore included in stage 1 of the classifier. AUCs for ependymoma against medulloblastoma (Table 3) showed a very high AUC for myo-inositol/total choline, and high AUCs for creatine/total choline and N-acetyl aspartate/total choline. Stage 2 was constructed using myo-inositol/total choline alone, with creatine/total choline and N-acetyl aspartate/total choline as checks to verify class assignment.

**Table 3:** Area under the receiver operator curve for prospective study of scanner-produced spectra of the three main cerebellar tumour types

Ratio	Pilocytic astrocytoma vs. all	Ependymoma vs. all	Medulloblastoma vs. all	Ependymoma vs. medulloblastoma
Cr/tCho	0.849	0.944	0.562	0.918
NAA/tCho	0.882	0.659	0.872	0.830
mIns/tCho	0.677	0.985	0.608	0.978
NAA/Cr	0.998	0.749	0.818	0.610
mIns/Cr	0.624	0.696	0.716	0.862
mIns/NAA	0.912	0.894	0.615	0.840
LMM 0.9/tCho	0.834	0.560	0.795	0.670
LMM 1.3/tCho	0.690	0.596	0.635	0.661
LMM 0.9/LMM 1.3	0.739	0.640	0.639	0.525

*Cr* creatine, *LMM* lipids and macromolecules, *mIns* myo-insoitol, *NAA* N-acetyl aspartate, *tCho* total choline

**Fig. 5:**
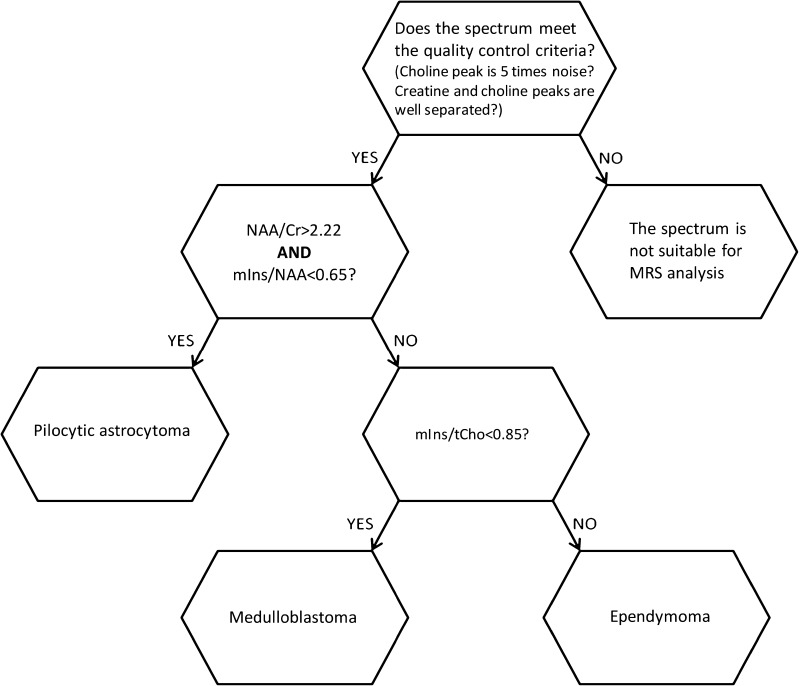
The new optimised classification decision scheme uses all data. *Cr* creatine, *mIns* myo-inositol, *MRS* magnetic resonance spectroscopy, *NAA* N-acetyl aspartate, *tCho* total choline

Cutoff values were determined for all these ratios using the following classification scheme (Fig. 5):

- Step 1: For a tumour to be assigned to the class of pilocytic astrocytoma, N-acetyl aspartate/creatine > 2.22, and myo-inositol/N-acetyl aspartate <0.65.
- Step 2: A tumour not classified as a pilocytic astrocytoma is assigned to the class of medulloblastoma if myo-inositol/total choline <0.85.

This updated scheme gave a correct classification rate of 90.6% for the 3 main tumour types, correctly classifying 48 of the 53 spectra (Table 4). Misclassifications include 1 ependymoma being classified as a medulloblastoma, 2 medulloblastomas as pilocytic astrocytomas, 1 medulloblastoma as an ependymoma and the outlier pilocytic astrocytoma as an ependymoma.

**Table 4:** Classification of cerebellar tumours using a multicentre dataset

Diagnosis	Number of patients	Correct classification	MR spectroscopy classification		
			Pilocytic astrocytoma	Ependymoma	Medulloblastoma
Pilocytic astrocytoma	16	94%	15	1	0
Ependymoma	8	88%	0	7	1
Medulloblastoma	29	90%	2	1	26

### Other tumours of the cerebellum

Tumours with diagnoses other than pilocytic astrocytoma, medulloblastoma or ependymoma form a diverse group. There were four tumour types in the cohort. Values for each of the ratios are shown in Table 5.

**Table 5:** Values for ratios in rare tumours of the cerebellum

Ratio	Atypical teratoid rhabdoid tumour (*n*=3)		Diffuse astrocytoma (*n*=1)	Ganglioglioma (*n*=1)	High-grade tumour (*n*=1)
	Mean	Standard error of the mean			
Cr/tCho	0.123	0.050	1.245	0.651	0.590
NAA/tCho	0.584	0.299	1.129	1.005	0.967
mIns/tCho	0.386	0.293	1.299	0.499	0.872
NAA/Cr	4.502	0.580	0.907	1.543	1.638
mIns/NAA	0.547	0.222	1.150	0.496	0.903
mIns/Cr	2.593	1.318	1.043	0.766	1.479
LMM 0.9/tCho	0.661	0.451	0.152	0.283	0.521
LMM 1.3/tCho	0.838	0.515	0.000	0.448	1.902
LMM 0.9/LMM 1.3	1.799	1.644	0.000	0.632	0.274

*Cr* creatine, *LMM* lipids and macromolecules, *mIns* myo-insoitol, *NAA* N-acetyl aspartate, *tCho* total choline

**Fig. 6:**
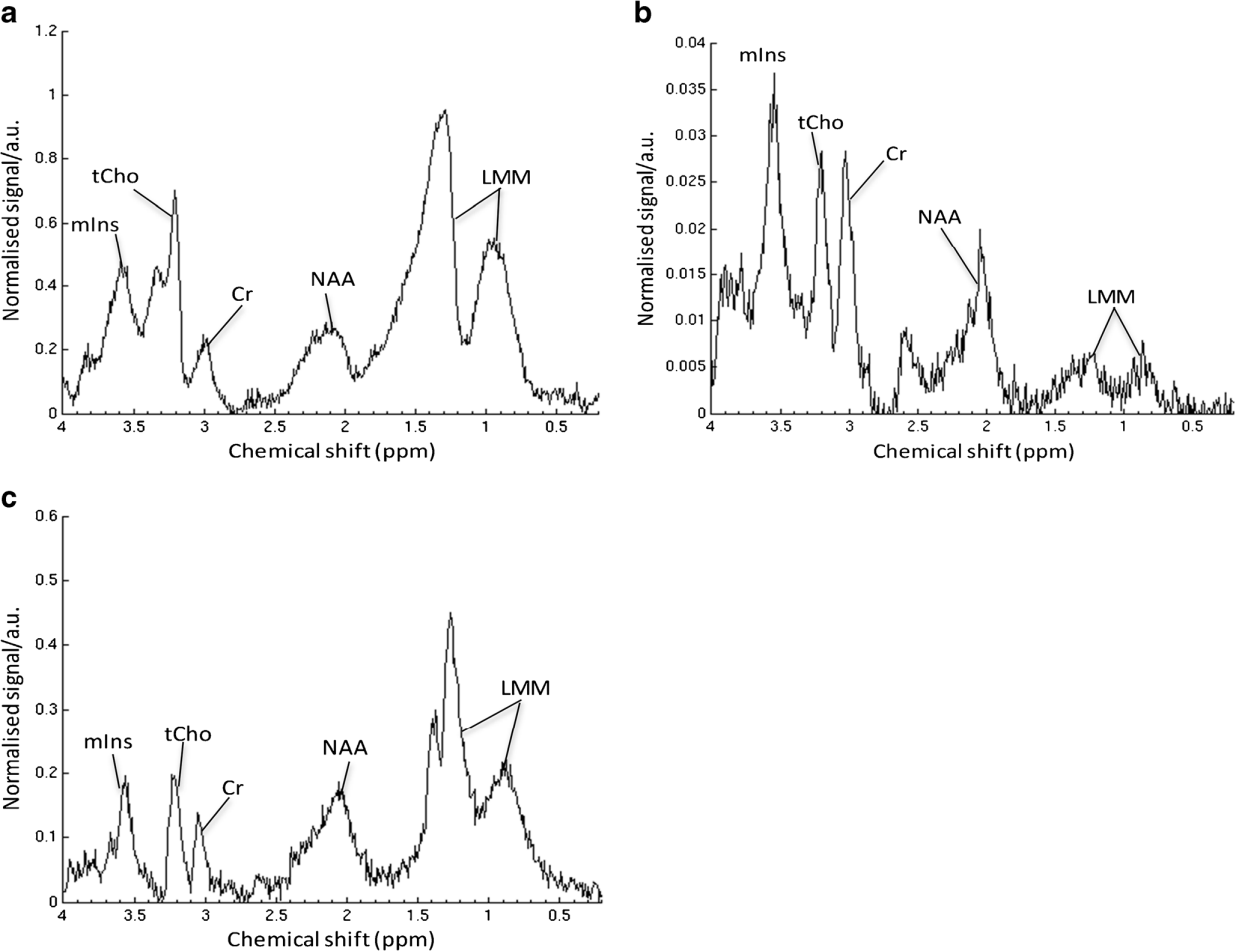
^1^H MR spectra from unusual main tumour types: (**a)** atypical teratoid rhabdoid tumour, (**b)** diffuse astrocytoma of the cerebellum and (**c)** high-grade lesion of the cerebellum. *a.u* arbitrary units, *Cr* creatine, *LMM* lipids and macromolecules, *mIns* myo-inositol, *NAA* N-acetyl aspartate, *ppm* parts per million, *tCho* total choline

There were two atypical teratoid rhabdoid tumours in the dataset; each of these had a different metabolite profile reflecting the large variability in metabolite ratios for this tumour group. The average N-acetyl aspartate/creatine is high due to the presence of a very small creatine peak in one of the cases, and would lead to classification as a pilocytic astrocytoma if the tumour classification scheme for the three most common tumours was used. Inspecting the spectra for atypical teratoid rhabdoid tumours confirmed no sharp peak at 2.0 ppm indicating the true N-acetyl aspartate level is very low (Fig. 6), which would be unusual for a pilocytic astrocytoma.

The diffuse astrocytoma spectrum (Fig. 6) exhibited a high myo-inositol peak, and thus showed high values for ratios containing myo-inositol. Although this tumour would be classified as an ependymoma using the updated peak height classifier, these values are still lower that those typically seen in ependymoma spectra. The spectrum of the diffuse astrocytoma also shows very small lipid and macromolecule peaks and a peak at 2.6 ppm likely due to citrate, which may help differentiate it from ependymoma.

The high-grade tumour has metabolite ratios close to the cutoff value for distinguishing between medulloblastoma and ependymoma spectra. The spectrum has features associated with high-grade tumours, in particular, high lipids (Fig. 6). It was not possible to obtain a definitive diagnosis for this tumour from the histopathology.

The ganglioglioma was classified as a medulloblastoma on step 2 of the updated peak height classifier. However, N-acetyl aspartate/total choline is much higher than would be expected for a medulloblastoma and the ratios myo-inositol/N-acetyl aspartate and myo-inositol/creatine are lower than in medulloblastoma.

## Discussion

A multicentre study was performed to evaluate a previously published diagnostic tool for classifying cerebellar tumours in children according to their MR spectroscopy peak height ratios [23]. A correct classification rate of 66% was obtained for tumours with a diagnosis of pilocytic astrocytoma, medulloblastoma or ependymoma using the original method. As this accuracy is lower than that reported for classifiers based on pattern recognition in adult brain tumours [2–4, 6, 21], it is important to explore whether there is an inherent weakness in the approach or whether the parameters used were poorly determined.

One weakness of the original analysis tool was that it was based on a small dataset. Whilst mean values for metabolite ratios can be calculated with some accuracy from small numbers of spectra, data variability is much more poorly determined. In this situation, the correct ratios to discriminate among tumours may be selected, but the optimum cutoff may be inaccurate. This problem is well demonstrated by the discrimination between pilocytic astrocytomas and the combined group of medulloblastomas and ependymomas. Although the ratio N-acetyl aspartate/creatine was significantly higher in pilocytic astrocytoma than the other tumours in the original dataset and remained so in the new dataset, several pilocytic astrocytomas in the new dataset had N-acetyl aspartate/creatine with lower values than the original cutoff of 4. Adjusting the cutoff to 2.2 allowed good discrimination between pilocytic astrocytoma and the combined group of medulloblastoma and ependymoma for the whole multicentre dataset, and this value is likely to be much better determined in the larger dataset.

Where there are very few spectra available for a given diagnosis, even mean values are poorly determined and an inappropriate variable can be selected for discrimination. This was the case for ependymomas in the original scheme. Altering the cutoff will not improve the accuracy in this situation; a more appropriate variable is required. When the most discriminatory metabolite ratio for ependymomas was determined from the larger dataset and used to refine the diagnostic tool, a classification rate of 90.6% was obtained, with 48 of 53 spectra correctly classified.

One of the greatest challenges in prospectively evaluating the diagnostic tool on multicentre data was including spectra acquired and processed on different scanners. Acquisition and processing differs among scanners, with varying amounts of baseline correction and level of water suppression leading to challenges in accurately measuring peak heights. Despite this, it was encouraging to see a classification rate for the 3 main tumours of greater than 90% with the updated scanner classifier, implying that straightforward peak measurement from scanner processed data can be used to analyse MR spectroscopy from multiple scanners.

A further benefit conferred from a larger cohort is assessment of rarer tumour types. Spectra for atypical teratoid rhabdoid tumours and the high-grade tumour showed a strong signal for lipids and macromolecules at 1.3 ppm in keeping with their poor prognosis [24]. The diffuse astrocytoma spectrum closely resembled the ependymoma spectra due to the presence of a high myo-inositol peak. This peak would be in keeping with observations made in diffuse gliomas [25] and in grade 2 gliomas [26, 27], all of which noted a strong signal from this metabolite in keeping with glial lineage. Classifying small numbers of many different tumour types is a challenge for any classification scheme, particularly for a simple rule-based classifier. We were, however, able to identify specific changes in spectra from rare tumours that not only provided a unique identifier, but were also consistent with known biological characteristics of the tumours. Despite providing interesting observations, it is difficult to draw firm conclusions from data on rare cerebellar tumours. Although it may be relatively straightforward to differentiate some rare lesions from other more common tumours (e.g., atypical teratoid rhabdoid tumour from pilocytic astrocytoma) on conventional imaging, others are characterised by overlapping imaging characteristics making them difficult to diagnose conclusively without additional information. MR spectroscopy may be a helpful addition to standard sequences in differentiating atypical teratoid rhabdoid tumour or ependymoma from medulloblastoma. It is important to remember that MR spectroscopy is not interpreted in isolation: rather, it is intended to enhance conventional MRI reporting to facilitate diagnosis in ambiguous cases.

Although short echo time MR spectroscopy provides more information on additional metabolites than long echo time MR spectroscopy, there are difficulties associated with signals from lipids and macromolecules. The frequent presence of a broad peak encompassing the region containing N-acetyl aspartate (around 2.0 ppm) can make this metabolite difficult to quantify. Using a “peak fitting” method (calculating metabolite concentration from the area under the peak) in the original dataset attributed N-acetyl aspartate to the entire broad peak, considerably overestimating N-acetyl aspartate peak area. In this study, the height of the narrow peak at around 2.0 ppm was used for N-acetyl aspartate with the height of the broad spectrum at 2.0 ppm used in cases where no narrow peak was present. Although N-acetyl aspartate peak height values still incorporate some macromolecular signal, they have a smaller overestimation than peak area. This does not affect the validity for classification, but should be considered when drawing biological conclusions from N-acetyl aspartate values.

Although 1.5-T single-voxel spectroscopy remains a commonly used method, 3-T single-voxel spectroscopy is becoming increasingly used. Whilst the general approach used in this paper is applicable to 3-T single-voxel spectroscopy, the specific algorithm would need to be generated and validated on 3-T data. In particular, multiplet peaks such as myo-inositol have a different appearance in 3-T compared with 1.5-T single-voxel spectroscopy. The current method could, however, be applied directly to data acquired using 1.5-T multi-voxel MR spectroscopy and may identify areas that are most representative of tumour.

Results of this study correlate with findings of previous studies and are biologically plausible in the context of tumour behaviour. Our finding of high N-acetyl aspartate/creatine in pilocytic astrocytoma is in accordance with reports from several studies in adults that found this ratio to be high in low-grade tumours with a good prognosis [2, 6, 28]. Evidence suggests N-acetyl aspartate/creatine is a good discriminator between high- and low-grade childhood tumours [29]. N-acetyl aspartate is a marker of neuronal integrity often considered to indicate normal brain tissue, with decreased levels in spectra of more highly malignant brain tumours [5, 7, 23, 30, 31]. Medulloblastoma and ependymoma had lower N-acetyl aspartate/creatine, in accordance with reports of low N-acetyl aspartate in malignancy and as a significant discriminator between ependymoma and other tumours [10]. This finding is also in agreement with reports of significantly lower levels of creatine in pilocytic astrocytoma compared to other tumour types [10]. Creatine, present in neuronal and glial cells, is involved in energy storage and metabolism and although this metabolite is stable in normal brain, levels vary in pathological states. The mean creatine of untreated tumors has been found to be half that of normal brain tissue both in vivo and in vitro [32] with reports of decreased creatine levels in gliomas [10, 11]. Concentrations of this metabolite may be related to tumour cell type rather than grade as no pattern of reduced or increased creatine has been reported in more malignant tumours [10].

The low myo-inositol/N-acetyl aspartate found in pilocytic astrocytoma is consistent with reports of pilocytic astrocytoma exhibiting low myo-inositol with a significantly lower myo-inositol/total choline than other tumours [10]*.* The role of myo-inositol is poorly understood*,* although interestingly this metabolite has been found to be present in high concentration in other astrocytoma types supporting the theory of myo-inositol being an astrocyte marker [33]. Our finding of high myo-inositol/total choline in ependymoma agrees with earlier reports of high myo-inositol in ependymoma [33]. In this study, myo-inositol was particularly high in grade II ependymoma. The low myo-inositol/total choline in medulloblastoma in this study is in accordance with findings of particularly high total choline levels in medulloblastoma [10]. Choline is involved in membrane synthesis and breakdown, increasing with cell proliferation in highly malignant tumours [5, 18, 34].

Diagnosing paediatric brain lesions is complex and continually evolving, with the advent of molecular subtyping providing more detailed classification of cerebellar tumours. According to the most recent World Health Organisation classification [35], medulloblastomas may be classified into four distinct subtypes corresponding to molecular characteristics. The histopathological classification of medulloblastoma has, however, been retained and is in continued use in clinical practice. Histopathological and molecular classifications may be used independently. Although molecular subtyping may influence treatment strategy and intensity in the future, research into the best way to use this information to determine management is ongoing and medulloblastoma is generally not yet treated according to genetic profile outside a clinical trial. As this was a relatively small, multicentre study with samples collected over a long period of time, molecular data were unavailable precluding further exploration at this stage. Determining molecular subtype noninvasively is an important goal and there is already published evidence that this can be achieved to some extent by both standard MRI and MR spectroscopy [36, 37]. A larger study would be necessary to map MR spectroscopy peak heights to enable diagnosis of different genetic profiles. It would be interesting to explore the role of MR spectroscopy and peak height measurement further as evidence for treatment according to novel diagnostic criteria evolves.

The tumour classifier is a simple and fast way of assessing a spectrum and is ideal in a clinical scenario when sophisticated analysis tools are not available. The modified classifier described offers a high classification rate between the three main types of cerebellar tumours. This scheme does not incorporate information from the whole set of metabolites detected, which could aid in classifying both common and rarer tumours. Incorporating this information in a quantitative method would, however, require more sophisticated analysis tools currently not readily accessible in routine clinical practice.

## Conclusion

A previously published tool for aiding the diagnosis of childhood cerebellar tumours using peak height measurements of MR spectroscopy data was evaluated prospectively on multicentre data and found to have only moderate accuracy. A tool developed from an expanded dataset resulted in accurate classification, implying that sample size is an important prerequisite. This new noninvasive diagnostic aid may provide accurate results from MR spectroscopy peak height measurement across different scanners. Prospective evaluation should be conducted to determine the true accuracy of this promising diagnostic tool.
